# Climate Change Sensitivity Index for Pacific Salmon Habitat in Southeast Alaska

**DOI:** 10.1371/journal.pone.0104799

**Published:** 2014-08-15

**Authors:** Colin S. Shanley, David M. Albert

**Affiliations:** The Nature Conservancy, Juneau, Alaska, United States of America; University of Vigo, Spain

## Abstract

Global climate change may become one of the most pressing challenges to Pacific Salmon conservation and management for southeast Alaska in the 21^st^ Century. Predicted hydrologic change associated with climate change will likely challenge the ability of specific stocks to adapt to new flow regimes and resulting shifts in spawning and rearing habitats. Current research suggests egg-to-fry survival may be one of the most important freshwater limiting factors in Pacific Salmon's northern range due to more frequent flooding events predicted to scour eggs from mobile spawning substrates. A watershed-scale hydroclimatic sensitivity index was developed to map this hypothesis with an historical stream gauge station dataset and monthly multiple regression-based discharge models. The relative change from present to future watershed conditions predicted for the spawning and incubation period (September to March) was quantified using an ensemble global climate model average (ECHAM5, HadCM3, and CGCM3.1) and three global greenhouse gas emission scenarios (B1, A1B, and A2) projected to the year 2080. The models showed the region's diverse physiography and climatology resulted in a relatively predictable pattern of change: northern mainland and steeper, snow-fed mountainous watersheds exhibited the greatest increases in discharge, an earlier spring melt, and a transition into rain-fed hydrologic patterns. Predicted streamflow increases for all watersheds ranged from approximately 1-fold to 3-fold for the spawning and incubation period, with increased peak flows in the spring and fall. The hydroclimatic sensitivity index was then combined with an index of currently mapped salmon habitat and species diversity to develop a research and conservation priority matrix, highlighting potentially vulnerable to resilient high-value watersheds. The resulting matrix and observed trends are put forth as a framework to prioritize long-term monitoring plans, mitigation experiments, and finer-scale climate impact and adaptation studies.

## Introduction

Pacific Salmon (*Oncorhynchus* spp.) are a key cultural [1], ecological [2], and economic [3] driver in southeast Alaska with global socio-ecological value [4], [5]. In a recent study by the U.S. Forest Service, 96% of Alaskans said that salmon are essential to the Alaskan way of life [6]. More than 50 species of animals feed on spawning salmon each year, and 1 in 10 jobs in southeast Alaska is supported by salmon [6]. Yearly salmon productivity has varied considerably by species and watershed—the regional productivity has remained relatively resilient [3], although the influence of hatchery enhancements on wild salmon stocks is of concern [7]–[9]. Sustained production and harvest opportunities are likely the result of a portfolio effect [10] whereby thousands of watersheds and tens-of-thousands of streams provide a diversity of freshwater habitats, promoting phenotypic diversity, which buffers regional salmon returns against variability in ocean [11], near-shore [12], and freshwater conditions [13]. Looking to the future, predicted hydrologic change associated with climate change may very well present the biggest challenge to Pacific Salmon conservation and management in the 21^st^ Century [13]–[20].

Southeast Alaska is predicted to experience the largest change in winter days above freezing in all of North America due to climate change [21]. While boreal and arctic Alaska is expected to see the largest changes in absolute temperature [22], a small increase in temperature in southeast Alaska could have transformative ecological effects (e.g., [23]–[27]). The mean winter temperature is currently near freezing at −4°C [28]; therefore, with a relatively small increase in temperature, many watersheds and portions of sub-basins will no longer receive winter precipitation as snow, and will transition into rain-fed systems. Many watersheds will likely see decreased total snowpack in headwaters that have provided water storage and moderated flows for salmon streams throughout the summer, and maintained cooler summer stream temperatures [17], [25], [26].

Watersheds of southeast Alaska can be generally categorized into three hydrologic types: (1) rain-fed, (2) snow-fed, and (3) glacial [29]. Each hydrologic type is expected to exhibit a spectrum of change associated with climate change [17], [18], [29] and provide unique challenges to resident salmon population adaptation [16], [30]. In general, rain-fed systems will likely see increased winter flows, reduced summer flows, and higher stream temperatures all year [16], [17], [19], [20]. Snow-fed systems will likely see more variable discharge patterns with increased rain-on-snow events that can cause flooding, an earlier spring melt, and some may transition into rain-fed hydrologic types due to loss of adequate headwater snowpack [17], [29], [31]. Glacial systems will likely see increased discharge year-round (until some systems lose glaciers altogether), generally colder summer stream temperatures while glaciers are still present, and warmer winter water temperatures [24], [32]–[35].

Pacific Salmon are affected by hydroclimatic factors at every stage of their lifecycle. Starting with egg incubation in freshwater habitats, water temperatures have the greatest effect on development rates [36]. In addition, winter flow extremes can scour salmon eggs from mobile spawning substrates (i.e., smaller gravels) and cause increased sedimentation that eliminates or degrades habitat by reducing oxygenation [19]. Emergent fry and juvenile salmonids require adequate stream flows to maintain high quality, protected off-channel habitats in both summer and winter to support growth and survival [36]. Smolt migration requires adequate stream flows and appropriate temperature cues to reach estuarine rearing habitats in synchrony with high food availability in the near-shore marine environment [37], [38]. Development and spawning success during the adult life-phase is in-turn dependent on global ocean circulation patterns, feed availability, and adequate stream flows and suitable temperatures for return migration and spawning [36].

A review of the current literature suggests egg-to-fry lifestage survival under projected climate change may be one of the most important limiting factors for salmon productivity and conservation in southeast Alaska freshwater habitats [15], [18], [19], [27], [29]. Recent studies in Washington State have detailed information on air-to-stream water temperature relationships [19], extreme flow events, and survival estimates [15]; population modeling studies thus far suggest egg-to-fry survival may be a key limiting factor under a range of climate change scenarios for the Pacific Northwest [15]—in addition to a suite of temperature related factors that may be less of a concern to salmon in northern parts of their range [17]. However, summer habitat conditions warrant further research in southeast Alaska [17], [38]. Washington State also has generally larger river systems that are more affected by climate model predictions for drier, hotter summers for interior parts of the state [19]. Southeast Alaska's mostly small and steep watersheds may behave more like the Olympic Peninsula watersheds where the scouring of eggs due to winter flooding events is currently thought to be more of concern than summer habitat conditions (e.g., [27]). Warmer winter water temperatures in southeast Alaska may accelerate salmon development rates, increase off-channel rearing habitats, and improve productivity in some watersheds (e.g., moderately glacial systems) in northern parts of their range [17], [36], [38]; this has been evidenced by increased salmon catch records during the warm-phase of the Pacific Decadal Oscillation in Alaska (PDO; [26]). However, depending on specific watershed physiographic characteristics (e.g., mean elevation, slope, and floodplain viability), stream channel geomorphology, genetic diversity, and capacity for phenotypic plasticity [16], [20], [30], [39], directionally changing flow regimes will likely transition into less favorable habitats due to more frequent scouring events in segments of some watersheds [15].

Two primary questions were asked in this paper: (1) effects of future projection trends in temperature and precipitation on seasonal discharge patterns across southeast Alaska, and (2) vulnerability or resilience of watersheds to hydrologic change in relation to the current distribution of high-value salmon habitat. The project was conducted in five phases: (1) development of a comprehensive historical stream gauge station database for the region; (2) development of a transboundary geospatial database of watershed physiographic and climatic characteristics for historical and projected temperature and precipitation; (3) building and testing multiple regression-based monthly discharge models using AIC model selection; (4) mapping the regional discharge model results for projected change during the spawning and incubation period (September to March), and; (5) combining a climate change sensitivity index with an index of current salmon habitat and species diversity to develop a research and conservation priority matrix.

## Methods

### Historical discharge patterns

A database of historical stream gauge stations was developed for southeast Alaska using USGS mean monthly gauge station records and USGS gauge station catchment polygons. For gauge stations where a catchment polygon was not available, watersheds were delineated using the Shuttle Radar Topography Mission (SRTM) digital elevation model (DEM; 25 m) and the ArcGIS 10.1 Watershed delineation tool (ESRI, Redlands, CA). An inventory of all gauge stations ever recorded via the USGS National Water Inventory System was conducted; only those gauge stations without human alteration (e.g., dams, hydro plants, etc.) were obtained for analysis. When more than one gauge station existed in a watershed, the station with the longest period of record was selected. In order to achieve a spatial distribution necessary for landscape modeling, ≥5 years of monthly discharge data was used as a cut-off for modeling purposes. Furthermore, data post-1976 Pacific Decadal Oscillation (PDO) was also used as a cut-off so as not to confound the analysis with PDO cycles [13], [40]. Monthly means (cubic feet per second) for each gauge station across the period of record available were calculated for model development.

### Watershed physiography and climatology

A transboundary geospatial database of physical watershed characteristics, and historical and projected climatologies were developed to model present and future patterns of stream discharge for all watersheds. The SRTM DEM was used for the basis of analysis in all U.S. watersheds, and U.S. portions of transboundary rivers. The best available DEMs were used for Canadian reaches (Table 1). The climate modeling software ClimateWNA 4.62 [41] was used at a 1 km spatial resolution to map gridded estimates of monthly temperature and precipitation from the PRISM climate model [42]–[44]. The three top performing global climate models for the region (ECHAM5, HadCM3, and CGCM3.1; [22], [45]) from the Intergovernmental Panel on Climate Change (IPCC) Fourth Assessment were averaged into a single ensemble model for analysis. This ensemble model was used to project temperature and precipitation for the year 2080 using three global greenhouse gas emission scenarios to identify regional trends [41], [46]: B1 (low growth), A1B (moderate growth), A2 (high growth). The National Hydrography Dataset (NHD) was used to delineate lake coverage within the U.S., and the best available datasets were used for Canada (Table 1). Glacier coverage for all watersheds was delineated with the Alaska Department of Natural Resources 1∶2,000,000 glacier coverage. The transboundary watershed polygons (n = 1784) developed by the U.S. Forest Service were used to create a regional watershed database. Mean values were calculated for temperature, precipitation, and elevation. Percent coverage was calculated for lakes and glaciers. Values for the historical gauge station catchment polygons and the complete regional watershed polygons were calculated separately.

**Table 1:** Climatic and physiographic geospatial data sources used to develop multiple regression-based monthly discharge models for southeast, Alaska, USA.

Variable	Description
Basin Area	USGS gauge station catchment polygons were used for the historical gauge station analysis. The USFS transboundary watershed layer, derived from primarily USGS HUC10 polygons, was used for the regional analysis.
Precipitation	ClimateWNA version 4.62 [41] 1 km downscaling of PRISM climate model [42] with monthly means generated from the period 1961–1990.
Temperature	ClimateWNA version 4.62 [41] 1 km downscaling of PRISM climate model [42] with monthly means generated from the period 1961–1990.
Elevation	Alaska: Shuttle Radar Topography Mission DEM (25 m), British Columbia: Terrain Resource Information Management Program DEM (25 m), Yukon: Department of Environment DEM (70 m). These DEMs were combined and resampled to 70 m.
Lakes	Alaska: National Hydrography Dataset, British Columbia: British Columbia Watershed Altus, Yukon: North American Water Polygons by ESRI. These polygons were combined into one lake coverage.
Glaciers	Alaska Department of Natural Resources 1∶2,000,000 glacier coverage.

### Discharge models and sensitivity index

A hierarchy of multiple regression models aimed at explaining monthly discharge using six potential explanatory variables were tested (Table 2). These models were tested using multiple regression and AIC model selection criterion [47]. Seven *a priori* models were developed, starting with the simplest model where basin area is the only explanatory variable, to more complex models incorporating climatology and physiographic setting. The same models were tested for each month for consistency and comparability. The most parsimonious, best-fit models with the lowest ΔAIC scores and highest weights were selected for an accuracy evaluation and the final hydroclimatic sensitivity index.

**Table 2:** Differences in AIC scores (ΔAIC), weights (AIC*w*), and number of model parameters (*k*) used to develop monthly multiple regression-based discharge models for southeast, Alaska, USA.

Models		Month					
		January		February		March	
Predictors	*k*	Δ AIC	AIC*w*	Δ AIC	AIC*w*	Δ AIC	AIC*w*
Area	1	69	0.000	79	0.000	63	0.000
Area + Precip	2	55	0.000	71	0.000	53	0.000
Area + Precip + Temp	3	6	0.024	13	0.001	9	0.006
Area + Precip + Temp + Elev	4	2	0.214	8	0.010	6	0.021
Area + Precip + Temp + Elev + Lakes	5	3	0.106	2	0.219	3	0.136
Area + Precip + Temp + Elev + Glac	6	0	0.477	3	0.159	1	0.338
Area + Precip + Temp + Elev + Glac + Lakes	7	2	0.180	0	0.611	0	0.499
		April		May		June	
Predictors	*k*	Δ AIC	AIC*w*	Δ AIC	AIC*w*	Δ AIC	AIC*w*
Area	1	62	0.000	39	0.000	56	0.000
Area + Precip	2	44	0.000	23	0.000	45	0.000
Area + Precip + Temp	3	0	0.529	24	0.000	36	0.000
Area + Precip + Temp + Elev	4	2	0.207	0	0.484	0	0.501
Area + Precip + Temp + Elev + Lakes	5	4	0.076	2	0.184	2	0.229
Area + Precip + Temp + Elev + Glac	6	3	0.136	1	0.243	2	0.185
Area + Precip + Temp + Elev + Glac + Lakes	7	5	0.052	3	0.089	4	0.086
		July		August		September	
Predictors	*k*	Δ AIC	AIC*w*	Δ AIC	AIC*w*	Δ AIC	AIC*w*
Area	1	60	0.000	53	0.000	36	0.000
Area + Precip	2	43	0.000	29	0.000	8	0.010
Area + Precip + Temp	3	27	0.000	13	0.001	10	0.004
Area + Precip + Temp + Elev	4	0	0.483	0	0.372	3	0.105
Area + Precip + Temp + Elev + Lakes	5	2	0.220	7	0.013	5	0.040
Area + Precip + Temp + Elev + Glac	6	2	0.193	0	0.343	0	0.467
Area + Precip + Temp + Elev + Glac + Lakes	7	3	0.104	1	0.272	0	0.375
		October		November		December	
Predictors	*k*	Δ AIC	AIC*w*	Δ AIC	AIC*w*	Δ AIC	AIC*w*
Area	1	47	0.000	60	0.000	60	0.000
Area + Precip	2	22	0.000	44	0.000	45	0.000
Area + Precip + Temp	3	0	0.406	0	0.325	8	0.009
Area + Precip + Temp + Elev	4	1	0.309	0	0.407	3	0.096
Area + Precip + Temp + Elev + Lakes	5	2	0.124	0	0.323	2	0.153
Area + Precip + Temp + Elev + Glac	6	3	0.115	0	0.330	0	0.431
Area + Precip + Temp + Elev + Glac + Lakes	7	4	0.046	2	0.186	1	0.311

The eight most regionally relevant discharge model variables used in Wiley and Curran [48] for the state of Alaska and conterminous basins in Canada were considered for modeling: basin area, main channel length, mean channel slope, mean basin elevation, % lakes, % forest, % glaciers, mean precipitation and mean temperature, as well as an estimate of precipitation as snow [44]. A Pearson's pairwise correlation analysis was conducted on the data matrix for each month to identify any collinearities (lrl≥0.7). The resulting dataset used for analysis was paired down to six key variables: basin area, mean basin elevation, % lakes, % glaciers, mean monthly precipitation, and mean monthly temperature. The physiographic variables (i.e., elevation, lakes, and glaciers) and monthly discharge values were normalized with log transformations [49].

The best-fit multiple regression equations for each month were first run on the historical gauge station database. A yearly hydrograph was plotted for a cross-section of rain-fed, snow-fed, and glacial systems for observed, predicted, and future projections for visual interpretation. The model accuracy was evaluated by comparing the observed monthly means with the predicted monthly means in a percent error matrix. The best-fit monthly regression equations were then run on the regional database to calculate monthly means for present and future conditions across all watersheds.

A hydroclimatic sensitivity index was calculated for all watersheds by averaging the percent change in predicted discharge (A1B emission scenario) from September to March when salmon eggs are in the gravel, and broken into a relative rank index by standard deviations from the mean. A salmon habitat and species diversity index was calculated using the 2012 Alaska Department of Fish and Game (ADF&G) Anadromous Waters Catalog (AWC). The AWC mapped presence of salmon species by stream reach was converted to the kilometers of salmon stream in each watershed, weighted by the number of species (≤6 including Pacific salmon and steelhead, *O. mykiss*), and scaled by the percent of total salmon streams in the region. The final priority matrix was the hydroclimatic sensitivity index and the salmon habitat and species diversity index split into four simple risk-value categories with median value cut-points.

## Results

**Figure 1:**
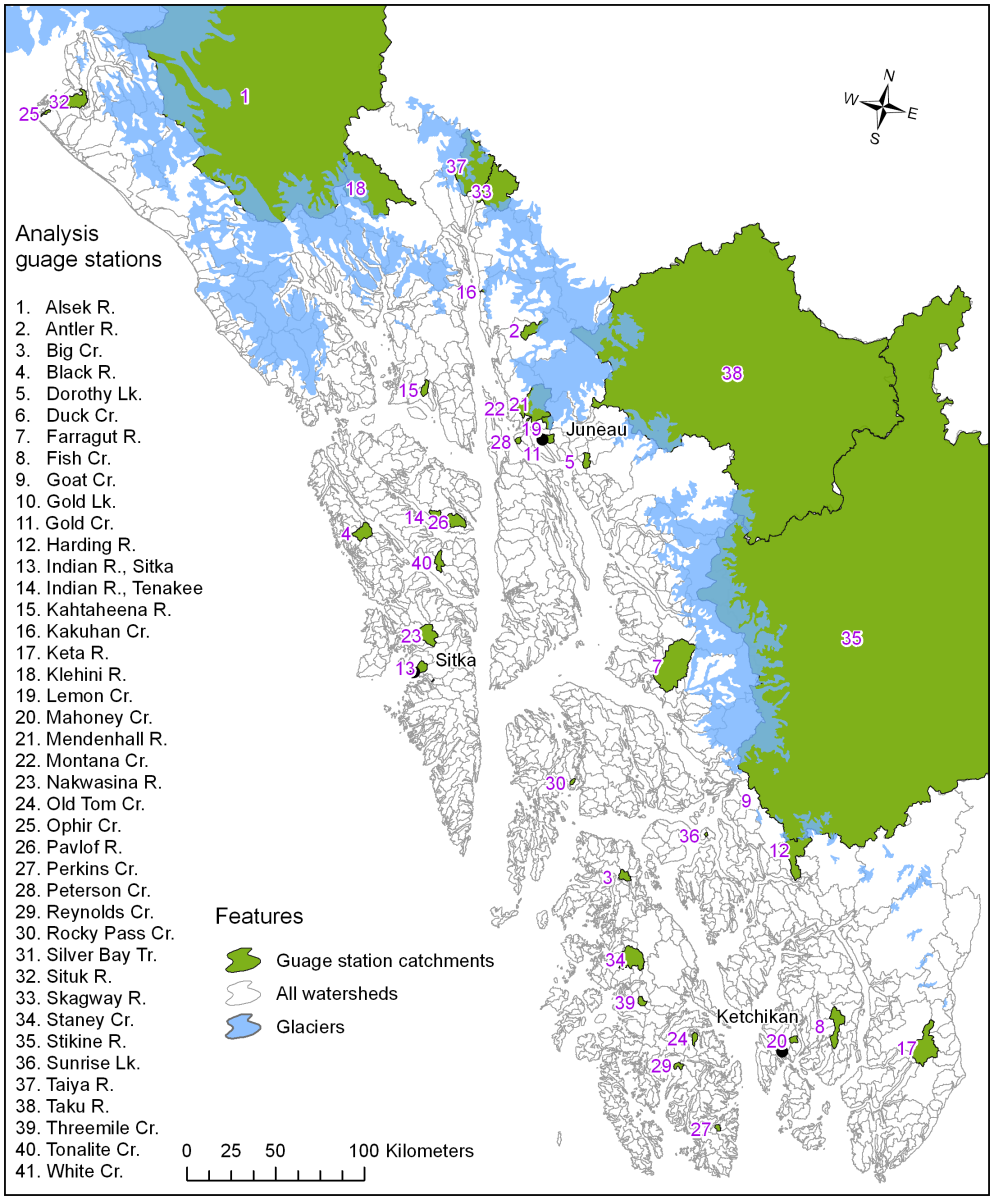
The watersheds of southeast, Alaska, USA, with the 41 gauge station catchments used for the development of regional multiple regression-based monthly discharge models.

The historical gauge station database (n = 41) showed a relatively well distributed spatial pattern with station locations spanning the latitudinal gradient of southeast Alaska from Ketchikan (55° latitude) in the south to Yakutat (59° latitude) in the north (Figure 1, Table S1). The gauge stations catchments were also distributed among islands (n = 18), mainland (n = 23), transboundary/interior (n = 3), and glacial systems (n = 13).

The AIC model selection process showed a characteristic seasonal pattern in variable selection. The simplest three variable models were in April and October with basin area, temperature and precipitation as the most parsimonious, best-fit model (Table 2). Elevation was selected as an additional variable for the early summer months of May, June, July, and again in the fall for the month of November (Table 2). Glaciers were added as a variable in August and September, and then again in December through March (Table 2). The most complex 6-variable model with the addition of lakes was selected in late winter months, February and March (Table 2). The regression coefficients matched seasonal patterns in discharge. Precipitation had a positive association in all models (Table 3). Temperature had a positive association in all months except for mid-summer, June, July and August (Table 3). Elevation had a positive association in summer months, May to September, and a negative association in winter months, November to March (Table 3). Glaciers had a positive association in summer months, August to September, and negative association in winter and spring months, November to March (Table 3). Lakes had a positive coefficient in all models in which it was included (Table 3). All models had high correlation values (Adjusted R^2^ = 0.964–0.980) with the best models in summer months (Table 3).

**Table 3:** Coefficients (β), standard error (SE), and adjusted R^2^ values for the most parsimonious multiple regression-based monthly discharge models for southeast, Alaska, USA.

	Constant	Area	Precip	Temp	Elevation	Glaciers	Lakes	Adj. R^2^
January	−5.410**	0.986**	0.002**	0.059**	−0.167**	−0.911*		0.967
	(0.269)	(0.032)	(0.0002)	(0.009)	(0.077)	(0.508)		
February	−6.327**	1.097**	0.002**	0.093**	−0.192**	−0.981*	2.765*	0.973
	(0.280)	(0.033)	(0.0003)	(0.011)	(0.073)	(0.522)	(1.361)	
March	−6.192**	1.036**	0.002**	0.094**	−0.154*	−1.146*	2.318	0.964
	(0.336)	(0.037)	(0.0004)	(0.015)	(0.079)	(0.571)	(1.501)	
April	−7.090**	1.054**	0.002**	0.134**				0.973
	(0.298)	(0.032)	(0.0003)	(0.015)				
May	−7.339**	0.979**	0.003**	0.068**	0.409**			0.980
	(0.432)	(0.030)	(0.0004)	(0.020)	(0.072)			
June	−6.923	0.946**	0.003**	−0.011	0.609**			0.980
	(0.576)	(0.031)	(0.0007)	(0.026)	(0.083)			
July	−6.210**	0.965**	0.002**	−0.068**	0.535**			0.978
	(0.668)	(0.033)	0.0007	(0.029)	(0.088)			
August	−6.299**	0.989**	0.002**	−0.039	0.322**	0.780		0.979
	(0.595)	(0.033)	(0.0005)	(0.025)	(0.081)	(0.615)		
September	−6.806**	0.991**	0.002**	0.043**	0.219**	1.129**		0.979
	(0.430)	(0.030)	0.0002	(0.018)	(0.073)	(0.530)		
October	−6.430**	1.029**	0.001**	0.070**				0.975
	(0.311)	(0.032)	(0.0001)	(0.013)				
November	−5.935**	1.031**	0.001**	0.068**	−0.106			0.972
	(0.291)	(0.032)	(0.0002)	(0.010)	(0.071)			
December	−5.492**	0.989**	0.001**	0.056**	−0.158**	−1.025**		0.967
	(0.295)	(0.033)	(0.0002)	(0.010)	(0.075)	(0.480)		

Standard errors are reported in parentheses.

*p<0.10,

**p<0.05.

**Figure 2:**
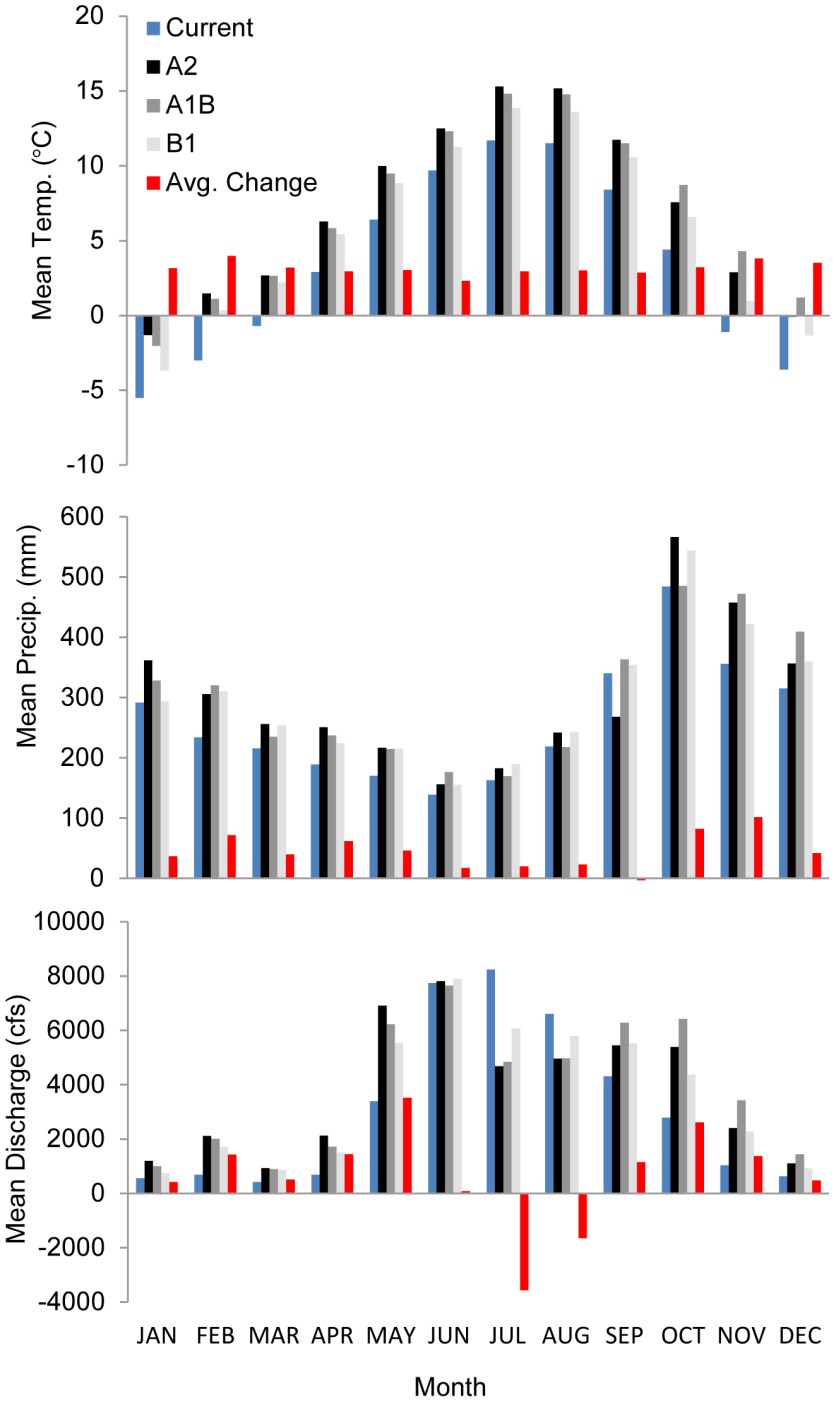
The historical mean (1961–1990) temperature (°C), precipitation (mm), and modeled discharge (cubic feet per second) for the 41 analysis gauge station catchments in southeast, Alaska, USA. These are shown next to projected changes for the year 2080 using an ensemble global climate model average (ECHAM5, HadCM3, and CGCM3.1) and three global greenhouse gas emission scenarios (B1, A1B, and A2) using multiple regression-based monthly discharge models.

Climate model projection trends for gauge station catchments in year 2080 show an increase in temperature, precipitation, and discharge, with the exception of July and August discharge decreases (Figure 2, Table S2, Table S3, Table S4, and Table S5). The mean monthly temperature for gauge station catchments is projected to increase from 3.4°C to an emission scenario range of 5.7–7.1°C, with the largest increases in late fall through spring (Figure 2). The mean monthly precipitation increased from 259.7 to 297.0–302.4 mm (14.4–16.4% change), with the largest increases in late fall (Figure 2). The mean monthly modeled discharge increased from 3088.3 to 3599.6–3905.0 cfs (16.6–26.4% change), with the greatest increases in early fall and spring (Figure 2).

**Figure 3:**
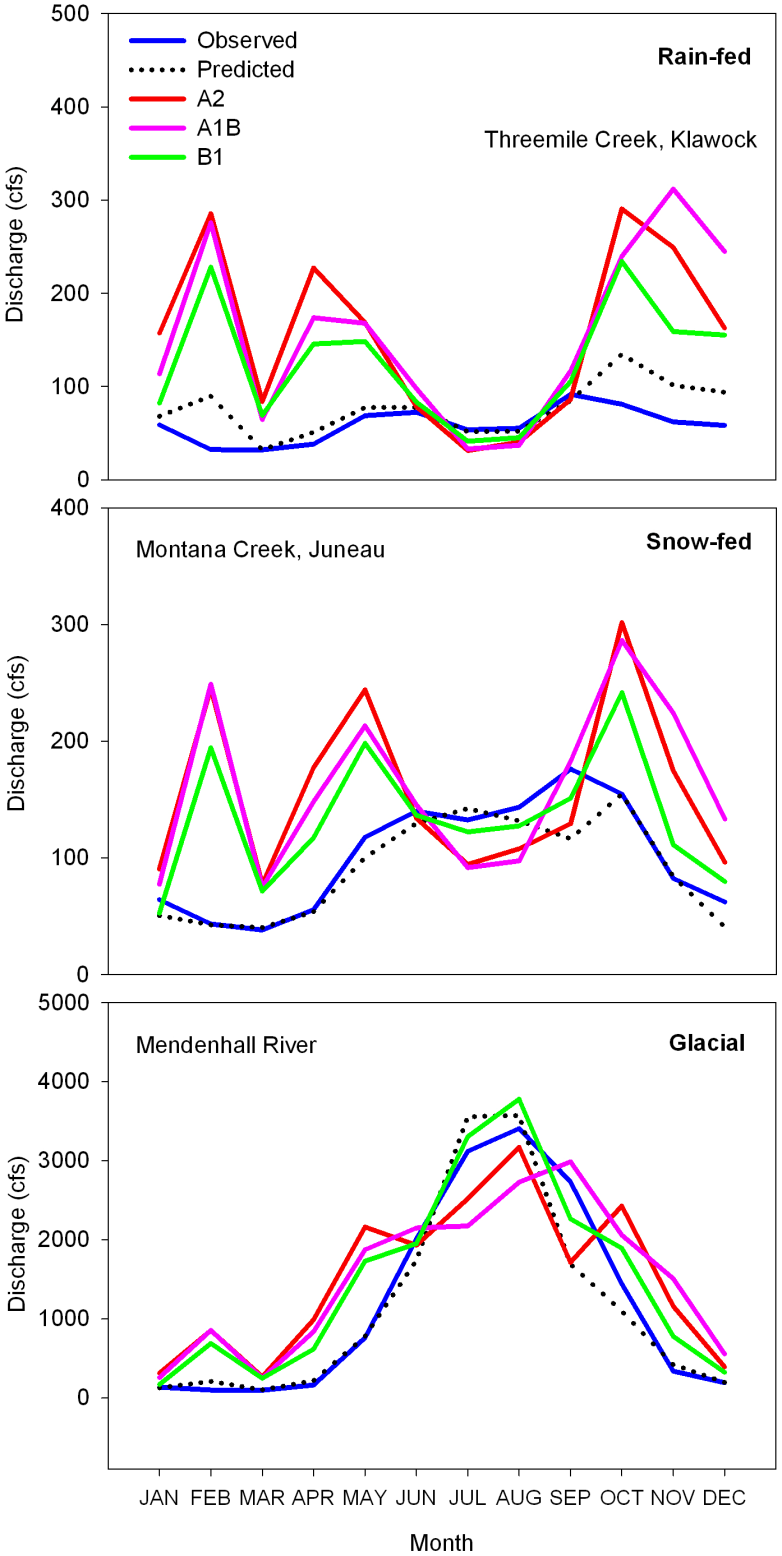
Yearly hydrographs of monthly means for a cross section of rain-fed, snow-fed, and glacial watersheds in southeast, Alaska, USA. Recorded observations, modeled predictions, and future projections for the year 2080 are plotted for comparison using multiple regression-based monthly discharge models and an ensemble global climate model average (ECHAM5, HadCM3, and CGCM3.1) run for three global greenhouse gas emission scenarios (B1, A1B, and A2).

The yearly hydrograph plots for a cross-section of rain-fed, snow-fed, and glacial systems showed characteristic seasonal discharge patterns (Figure 3). Threemile Creek near Klawock, on southern Prince of Wales Island, is a small rain-fed system that generally followed seasonal precipitation patterns with peak flows in the fall with little water storage (Figure 3). The projected hydrograph for Threemile Creek showed increased peak flows in the spring and fall, and similar or lower discharge in summer months (Figure 3). Montana Creek near Juneau is a medium-sized snow-fed system with discharge patterns that showed a spring melt and fall peak (Figure 3). The projected hydrograph for Montana Creek showed increased spring and fall peak flows, and lower discharge in summer months (Figure 3). The Mendenhall River near Juneau is a large glacial system with characteristic peak flows in mid-summer with a bell-shaped yearly hydrograph (Figure 3). The projected hydrograph for the Mendenhall River showed generally increased discharge year-round with a more spread out summer peak (Figure 3).

The historical gauge station model evaluation showed that monthly discharge was on average over predicted (Table S6). The mean monthly discharge absolute error for all models was 36.4%. The most accurate models were in summer months with a mean absolute error of 28.5%. The mean absolute error during the analysis period of September to March was 41.9%.

**Figure 4:**
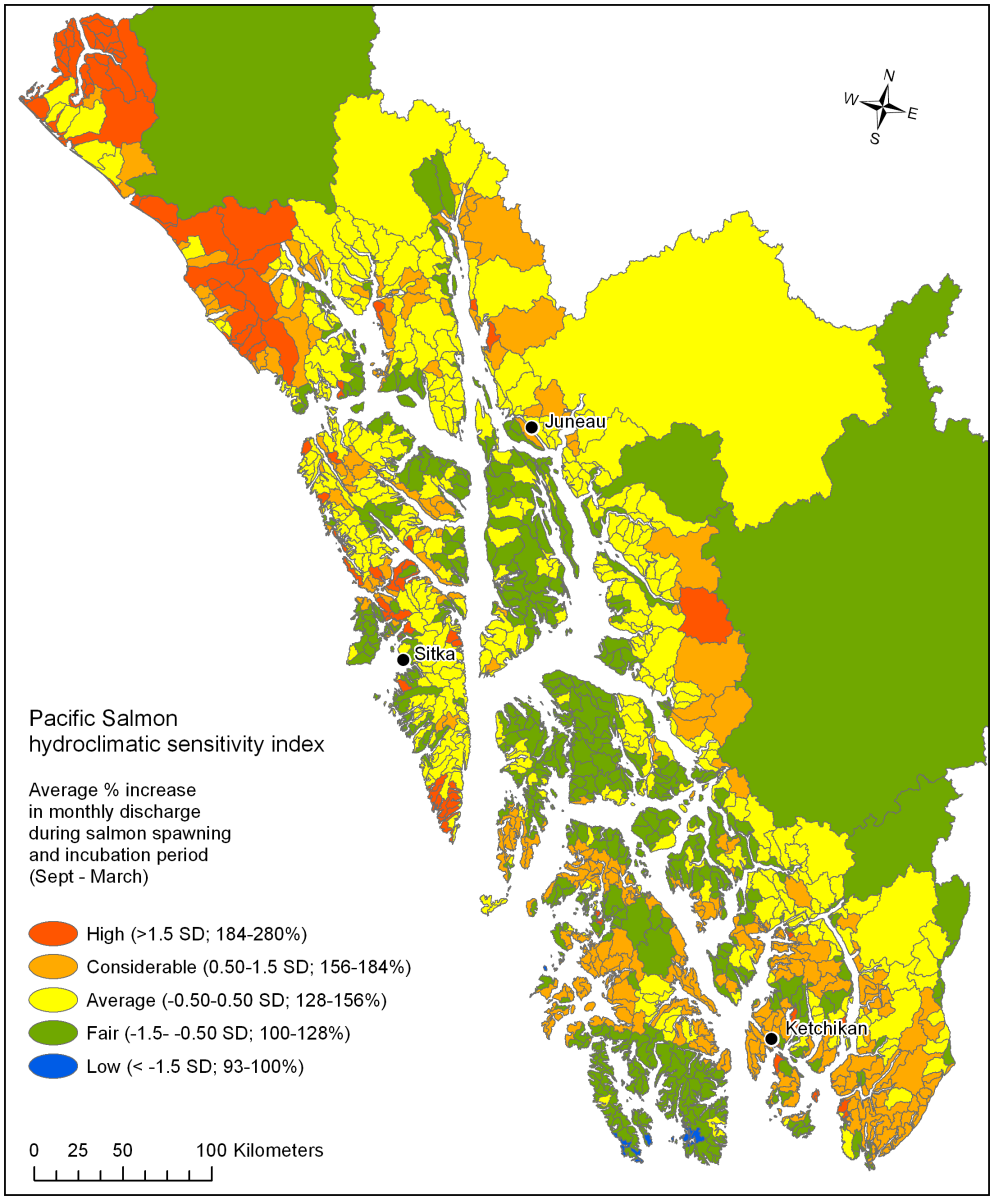
The watersheds of southeast, Alaska, USA (n = 1784) showing the Pacific Salmon hydroclimatic sensitivity index for predicted hydrologic change. This was derived using multiple regression-based monthly discharge models, an ensemble global climate model average (ECHAM5, HadCM3, and CGCM3.1) for temperature and precipitation, and the A1B global greenhouse gas emission scenario projections for the year 2080 to illustrate regional trends.

**Figure 5:**
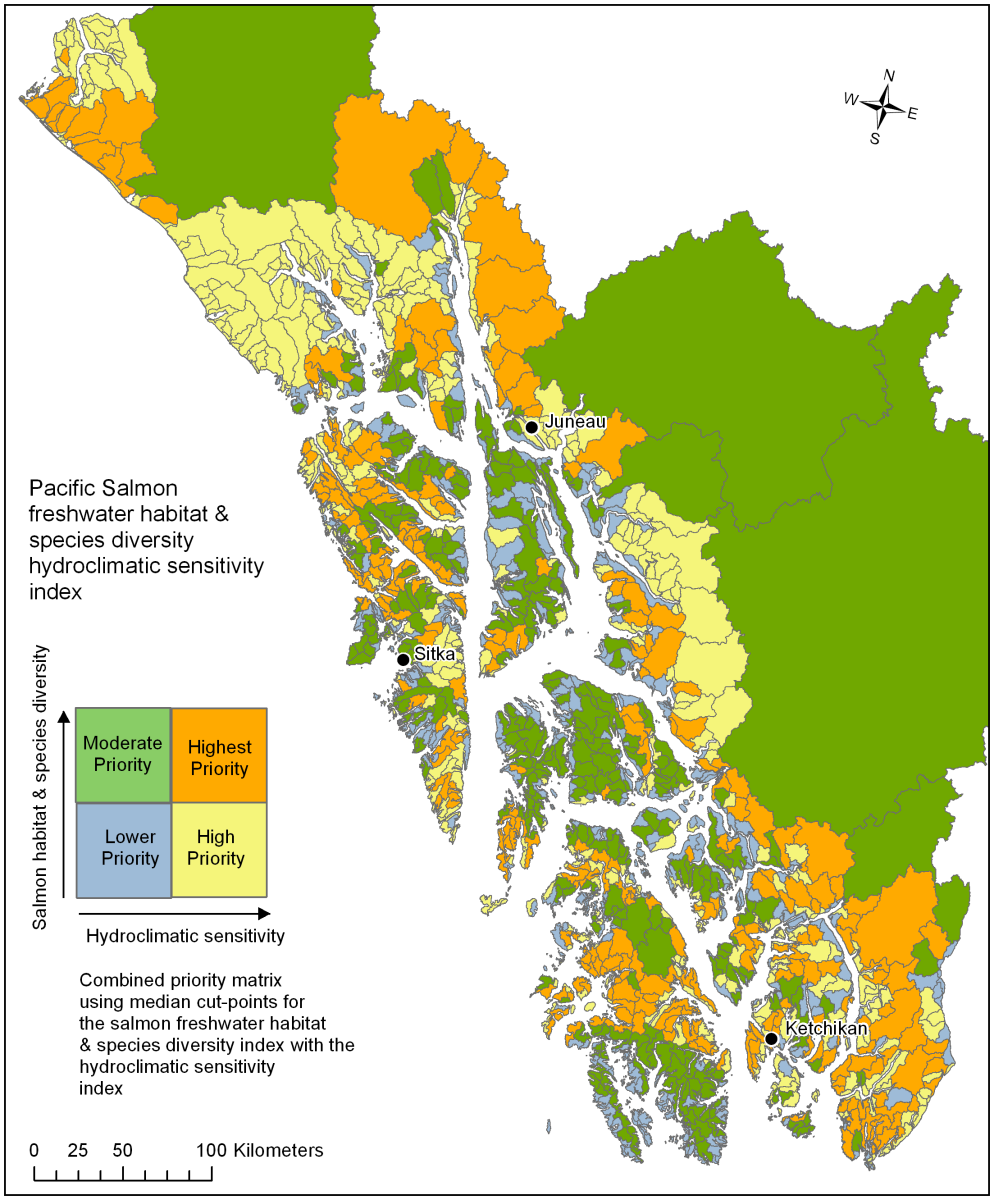
The watersheds of southeast, Alaska, USA (n = 1784) showing the Pacific Salmon freshwater habitat and species diversity index combined with the hydroclimatic sensitivity index developed in this study.

The regional hydroclimatic sensitivity index showed northern mainland and steeper, snow-fed mountainous watersheds with current winter temperatures close to freezing exhibiting the greatest change (Figure 4). The highest ranking group (>1.5 SD; 184–280%) were distributed along the northern mainland, including some glacial systems, and others were scattered throughout the region. Next, there was a group of watersheds with high spatial variability and moderate ranking (0.50–1.5 SD; 156–184%) with the highest concentration in the southeast corner of the region. Watersheds with average percent change (−0.50–0.50; 128–156%) occurred along the mainland, and included many glacial systems and mountainous areas of the major island groups. Fair to low ranking sensitivity (−0.50–<−1.5 SD; 93–128%) watersheds were located throughout the region in low elevation areas, and included several of the larger transboundary watersheds. The combined salmon habitat and species diversity hydroclimatic sensitivity index showed highest priority in systems that exhibited a combination of high salmon habitat and species diversity with changing hydrology (Figure 5).

## Discussion

The most striking result of the analysis was the transition in mean winter temperatures across the freezing threshold for many watersheds, and how it translated into changes in regional discharge patterns that are important for the reproduction and survival of Pacific Salmon in southeast Alaska. Combined with predicted increases in precipitation across all months, mean monthly discharge was forecasted to increase by approximately 1-fold to 3-fold during September to March when salmon eggs are in the gravel and exposed to more frequent scouring events. Plots of yearly hydrographs showed substantially increased peak flows in rain-fed and snow-fed systems in early spring and late fall across emission scenarios. The hydroclimatic sensitivity index showed the interaction between projected climate trends and the region's diverse physiography—a relatively predictable pattern that could be used as a preliminary framework to develop targeted long-term monitoring and potential mitigation strategies. The combined salmon habitat and species diversity sensitivity index showed clusters of high-value watersheds that could be prioritized for: (1) conservation of available genetic and life history diversity (e.g., run timing; [30]); (2) evaluation of local, reach-scale geomorphic sediment mobility and susceptibility to scour; (3) restoration to improve natural variability and general ecological resilience [15], [50]; (4) monitoring harvest pressures and escapement goals in light of new environmental factors, and; (5) developing finer-scale climate impact and salmonid adaptation studies (e.g., [19], [20], [27], [34], [38], [51]).

The ability of the discharge models to accurately reflect seasonal flow patterns among watersheds and watershed types is a positive indicator for the models' ability to predict regional trends in ungauged watersheds across the landscape. An evaluation of model selection, coefficient direction, and yearly hydrographs agreed with the current general understanding of regional hydrologic patterns [13], [24], [32], [52]. The models general over-prediction of discharge is common among discharge models [48]. The percent error scores (both positive and negative) are likely attributed to a combination of sources: resolution of the climate data; older and more remote faulty gauge station records, especially during winter months when stations are more prone to freezing issues, and; water storage ecological processes (e.g., wetlands) simply not captured in the generalized linear regional model. This regional modeling effort is an important step forward in the development of future predictive models for southeast Alaska. Uncertainty associated with the climate modeling and potential effects could be further reduced with higher spatial and temporal resolution datasets that can capture ecological processes such as geomorphologic change and generate flood frequency statistics. Future forecasting studies would also benefit from a broader stream gauge network that represents the spectrum of potentially vulnerable to resilient salmon producing watersheds, with an emphasis on monitoring extreme events such as the frequency of flooding, coupled with experimental studies on egg-to-fry survival and salmonid adaptive capacity.

Nested within the hydroclimatic sensitivity framework, river channel types will change with flow rates and different species of salmon will likely be affected differently based on their body size, freshwater life-history and residence time, which warrants further research. The body size of salmon species directly relates to the burial depth of eggs and therefore exposure to scouring events in mobile spawning substrates [53], [54]. The spawning location in watersheds directly relates to the rate of flow and sedimentation exposure [19]. The highest flow and sedimentation rates are generally expected in the central parts of the mainstem and significant tributaries, as opposed to the headwaters or floodplains [19]. Steelhead (*O. mykiss*) spawn in the spring within the highest reaches of the watershed [36] and could, for example, be less prone to winter souring events. Coho salmon (*O. kisutch*), sockeye salmon (*O. nerka*), and Chinook salmon (*O. tshawytscha*) spawn in the mid-portions of the watersheds, have longer residence times [36], and may therefore be the most exposed species to flooding events; however, their relative body sizes will help egg burial depths and exposure to scour [53]. Pink salmon (*O. gorbuscha*) and chum salmon (*O. keta*) spawn and rear in the lowest reaches of watersheds and migrate a few weeks after they emerge from the gravel (April–May) [36], and may therefore be less vulnerable to scouring where floodplains are intact. However, potential sea level rise is a factor for low-elevation pink salmon and chum salmon spawning habitat in southern southeast Alaska [17], [55].

Hydrologic projections for Washington State predict a complete loss of snow-fed hydrologic systems by the year 2080 under the same A1B emission scenario, with only a few watersheds in the North Cascade mountains retaining transitional rain/snow characteristics [18], [56]. The snow-fed watersheds are predicted to transition into rain-fed hydrologic patterns with low flows in the summer and more intense flooding events during the winter months [18]. Increases in temperature appear to have a stronger influence on flows than changes in precipitation in Washington State [19]; this also appears to be the case with the largest changes in projected flows for southeast Alaska occurring during months when many watersheds cross the freezing threshold. Thus far, simulation studies suggest low-elevation floodplain and wetland connectivity restoration efforts to slow increased flow rates and improve summer water storage are the most effective climate change mitigation strategies in population viability models for Washington State [15]. These ecosystem engineering techniques could be further tested in historically impacted watersheds in southeast Alaska [57].

The results of this analysis should be treated as a hypothesis of potential change, and a framework for finer-scale experimental studies that investigate the long-term effects of changing hydrologic regimes, inter-annual variability, extreme events, and salmonid adaptive capacity in southeast Alaska. As global climate models, hydrologic forecasting and downscaling techniques improve, the absolute values for projected temperature, precipitation, and stream discharge will change and model predictions will improve. The results of this study provide a compelling case for how climate change trends could interact with the regions diverse physiography in a relatively predictable pattern where research and mitigation experiments should be prioritized for effective salmon conservation investments.

## Supporting Information

Table S1 **Recorded mean monthly discharge (cubic feet per second) for 41 southeast, Alaska, USA gauge stations with ≥5 year period of record.**
(DOCX)

Table S2 **Multiple regression-based monthly discharge model predictions for 41 southeast, Alaska, USA gauge station catchments.**
(DOCX)

Table S3 **Multiple regression-based monthly discharge models for projected future discharge (cubic feet per second) for 41 gauged catchments in southeast, Alaska, USA using an ensemble global climate model average (ECHAM5, HadCM3, and CGCM3.1) and the B1 global greenhouse gas emission scenario for the year 2080.**
(DOCX)

Table S4 **Multiple regression-based monthly discharge models for projected future discharge (cubic feet per second) for 41 gauged catchments in southeast, Alaska, USA using an ensemble global climate model average (ECHAM5, HadCM3, and CGCM3.1) and the A1B global greenhouse gas emission scenario for the year 2080.**
(DOCX)

Table S5 **Multiple regression-based monthly discharge models for projected future discharge (cubic feet per second) for 41 gauged catchments in southeast, Alaska, USA using an ensemble global climate model average (ECHAM5, HadCM3, CGCM3.1) and the A2 global greenhouse gas emission scenario for the year 2080.**
(DOCX)

Table S6 **Multiple regression-based monthly discharge model percent error ((predicted-observed)/observed) for 41 southeast, Alaska, USA gauged catchments.**
(DOCX)
