# Correction: Climate Change Sensitivity Index for Pacific Salmon Habitat in Southeast Alaska

**DOI:** 10.1371/journal.pone.0112926

**Published:** 2014-11-03

**Authors:** The PLOS ONE Staff

There is an error in Table 3, “Coefficients (β), standard error (SE), and adjusted R2 values for the most parsimonious multiple regression-based monthly discharge models for southeast, Alaska, USA.” Please see the corrected Table 3 here.

**Table 3:** **Coefficients (β), standard error (SE), and adjusted R2 values for the most parsimonious multiple regression-based monthly discharge models for southeast, Alaska, USA.**

	Constant	Area	Precip	Temp	Elevation	Glaciers	Lakes	Adj. R^2^
January	−5.410**	0.986**	0.002**	0.059**	−0.167**	−0.911*		0.967
	(0.269)	(0.032)	(0.0002)	(0.009)	(0.077)	(0.508)		
February	−6.327**	1.097**	0.002**	0.093**	−0.192**	-0.981*	2.765*	0.973
	(0.280)	(0.033)	(0.0003)	(0.011)	(0.073)	(0.522)	(1.361)	
March	−6.192**	1.036**	0.002**	0.094**	−0.154*	-1.146*	2.318	0.964
	(0.336)	(0.037)	(0.0004)	(0.015)	(0.079)	(0.571)	(1.501)	
April	−7.090**	1.054**	0.002**	0.134**				0.973
	(0.298)	(0.032)	(0.0003)	(0.015)				
May	−7.339**	0.979**	0.003**	0.068**	0.409**			0.980
	(0.432)	(0.030)	(0.0004)	(0.020)	(0.072)			
June	−6.923**	0.946**	0.003**	−0.011	0.609**			0.980
	(0.576)	(0.031)	(0.0007)	(0.026)	(0.083)			
July	−6.210**	0.965**	0.002**	−0.068**	0.535**			0.978
	(0.668)	(0.033)	0.0007	(0.029)	(0.088)			
August	−6.299**	0.989**	0.002**	−0.039	0.322**	0.780		0.979
	(0.595)	(0.033)	(0.0005)	(0.025)	(0.081)	(0.615)		
September	−6.806**	0.991**	0.002**	0.043**	0.219**	1.129**		0.979
	(0.430)	(0.030)	0.0002	(0.018)	(0.073)	(0.530)		
October	−6.430**	1.029**	0.001**	0.070**				0.975
	(0.311)	(0.032)	(0.0001)	(0.013)				
November	−5.935**	1.031**	0.001**	0.068**	−0.106			0.972
	(0.291)	(0.032)	(0.0002)	(0.010)	(0.071)			
December	−5.492**	0.989**	0.001**	0.056**	−0.158**	−1.025**		0.967
	(0.295)	(0.033)	(0.0002)	(0.010)	(0.075)	(0.480)		

Standard errors are reported in parentheses.

*p<0.10, **p<0.05.
