# Supplementary material for: Climate Change Sensitivity Index for Pacific Salmon Habitat in Southeast Alaska
Source: PLoS One. 2014 Aug 15;9(8):e104799. doi: 10.1371/journal.pone.0104799 (PMC4134244; doi:10.1371/journal.pone.0104799)
Supplement: Table S1 — Recorded mean monthly discharge (cubic feet per second) for 41 southeast, Alaska, USA gauge stations with ≥5 year period of record. (DOCX) [file pone.0104799.s001.docx]

**Table S1.** Recorded mean monthly discharge (cubic feet per second) for 41 southeast, Alaska, USA gauge stations with ≥ 5 year period of record.

|  |  |  | Month | | | | | | | | | | | | Yearly Mean |
| --- | --- | --- | --- | --- | --- | --- | --- | --- | --- | --- | --- | --- | --- | --- | --- |
| Gauge Station | START | STOP | JAN | FEB | MAR | APR | MAY | JUN | JUL | AUG | SEP | OCT | NOV | DEC |  |
|  |  |  |  |  |  |  |  |  |  |  |  |  |  |  |  |
| ALSEK R NR YAKUTAT AK | 1991 | 2011 | 4986.1 | 4102.3 | 3926.6 | 6471.5 | 26136.5 | 70123.0 | 89136.7 | 79097.1 | 47719.5 | 23851.0 | 10380.1 | 7032.2 | 31080.2 |
| ANTLER R NR AUKE BAY AK | 1997 | 2011 | 36.2 | 28.9 | 25.1 | 45.4 | 165.9 | 302.2 | 260.5 | 220.8 | 214.4 | 159.1 | 77.3 | 58.1 | 132.8 |
| BIG C NR POINT BAKER AK | 1977 | 1981 | 67.9 | 82.5 | 70.8 | 92.1 | 56.4 | 44.7 | 28.1 | 41.2 | 100.0 | 254.0 | 150.1 | 80.5 | 89.0 |
| BLACK R NR PELICAN AK | 1977 | 1982 | 151.0 | 132.6 | 159.1 | 238.9 | 339.3 | 221.5 | 185.0 | 157.5 | 286.9 | 575.6 | 274.4 | 141.5 | 238.6 |
| DOROTHY LK OUTLET NR JUNEAU AK | 1986 | 2002 | 21.5 | 20.7 | 17.3 | 18.8 | 86.2 | 217.5 | 271.1 | 263.7 | 260.7 | 164.2 | 49.6 | 37.6 | 119.1 |
| DUCK C BL NANCY ST NR AUKE BAY AK | 1993 | 2004 | 3.2 | 2.7 | 2.5 | 2.8 | 2.5 | 2.0 | 2.5 | 3.7 | 7.7 | 9.0 | 4.9 | 5.3 | 4.1 |
| FARRAGUT R NR PETERSBURG AK | 1977 | 1993 | 794.8 | 648.4 | 560.4 | 755.0 | 1716.6 | 2463.7 | 2794.1 | 2780.6 | 2854.0 | 2591.0 | 1172.3 | 732.9 | 1655.3 |
| FISH C NR KETCHIKAN AK | 1977 | 2011 | 403.9 | 354.6 | 304.0 | 362.6 | 457.8 | 408.6 | 298.9 | 311.3 | 515.4 | 676.7 | 529.3 | 444.2 | 422.3 |
| GOAT C NR WRANGELL AK | 1977 | 1986 | 68.3 | 52.2 | 49.5 | 77.8 | 272.3 | 388.5 | 313.5 | 239.0 | 276.1 | 352.3 | 106.1 | 52.2 | 187.3 |
| GOAT LK OUTLET NR SKAGWAY AK | 1991 | 1997 | 1.5 | 1.8 | 1.5 | 1.4 | 19.1 | 43.2 | 31.7 | 21.2 | 18.0 | 10.9 | 3.1 | 1.9 | 12.9 |
| GOLD C NR JUNEAU AK | 1984 | 1997 | 25.0 | 24.9 | 22.0 | 41.6 | 138.8 | 200.0 | 182.0 | 162.0 | 179.4 | 160.5 | 58.9 | 44.0 | 103.3 |
| HARDING R NR WRANGELL AK | 1977 | 2004 | 317.1 | 251.8 | 232.7 | 417.2 | 941.6 | 1383.1 | 1266.4 | 1084.4 | 1214.8 | 1087.2 | 509.3 | 353.9 | 755.0 |
| INDIAN R NR SITKA AK | 1980 | 2006 | 94.4 | 79.5 | 59.6 | 66.1 | 99.6 | 82.4 | 62.1 | 86.3 | 171.3 | 178.4 | 116.6 | 106.6 | 100.2 |
| INDIAN R NR TENAKEE AK | 1977 | 1982 | 58.2 | 43.1 | 40.0 | 76.8 | 141.0 | 110.0 | 58.0 | 36.0 | 95.0 | 176.0 | 106.0 | 57.0 | 83.1 |
| KAHTAHEENA R NR GUSTAVUS AK | 1999 | 2004 | 35.0 | 27.0 | 19.0 | 34.0 | 83.0 | 82.0 | 55.0 | 53.0 | 99.0 | 88.0 | 54.0 | 56.0 | 57.1 |
| KAKUHAN C NR HAINES AK | 1997 | 2011 | 1.4 | 1.0 | 0.9 | 2.4 | 10.0 | 21.0 | 28.0 | 26.0 | 18.0 | 9.7 | 4.0 | 2.5 | 10.4 |
| KETA R NR KETCHIKAN AK | 1977 | 1984 | 408.6 | 293.5 | 305.0 | 468.5 | 1148.0 | 1219.0 | 854.0 | 773.0 | 958.0 | 1479.0 | 710.0 | 434.0 | 754.2 |
| KLEHINI R NR KLUKWAN AK | 1981 | 1993 | 285.1 | 242.3 | 222.4 | 389.5 | 1782.1 | 3729.8 | 4150.6 | 3203.8 | 1762.9 | 1322.9 | 517.7 | 375.3 | 1498.7 |
| LEMON C NR JUNEAU AK | 2002 | 2011 | 16.3 | 10.2 | 9.3 | 30.7 | 132.7 | 307.7 | 461.4 | 507.4 | 443.3 | 191.4 | 57.5 | 24.8 | 182.7 |
| MAHONEY C NR KETCHIKAN AK | 1977 | 1981 | 57.6 | 56.8 | 62.6 | 65.2 | 110.1 | 121.9 | 87.1 | 67.9 | 106.7 | 193.5 | 111.1 | 67.8 | 92.3 |
| MENDENHALL R NR AUKE BAY AK | 1977 | 2011 | 135.6 | 100.1 | 96.9 | 162.7 | 762.0 | 2011.5 | 3119.9 | 3407.2 | 2733.8 | 1442.8 | 338.6 | 192.7 | 1208.6 |
| MONTANA C NR AUKE BAY AK | 1983 | 2011 | 64.0 | 43.3 | 38.1 | 55.6 | 117.8 | 140.0 | 132.5 | 143.5 | 176.1 | 154.6 | 82.3 | 62.2 | 100.8 |
| NAKWASINA R NR SITKA AK | 1977 | 1982 | 156.0 | 140.0 | 100.3 | 152.6 | 332.6 | 474.0 | 389.4 | 293.7 | 426.0 | 627.9 | 298.0 | 156.3 | 295.6 |
| OLD TOM C NR KASAAN AK | 1977 | 2011 | 63.5 | 53.4 | 51.1 | 52.1 | 38.3 | 21.9 | 11.9 | 16.6 | 37.9 | 73.3 | 76.8 | 69.1 | 47.2 |
| OPHIR C NR YAKUTAT AK | 1991 | 2011 | 18.2 | 15.3 | 13.4 | 12.6 | 12.7 | 5.8 | 3.9 | 8.7 | 19.1 | 30.8 | 25.2 | 22.9 | 15.7 |
| PAVLOF R NR TENAKEE AK | 1977 | 1981 | 146.3 | 122.6 | 107.2 | 180.4 | 198.5 | 148.2 | 114.1 | 90.2 | 214.5 | 506.9 | 209.9 | 78.8 | 176.5 |
| PERKINS C NR METLAKATLA | 1977 | 1993 | 53.0 | 42.8 | 40.6 | 35.5 | 24.3 | 14.4 | 11.3 | 15.6 | 33.6 | 57.7 | 69.3 | 50.4 | 37.4 |
| PETERSON C BL NF NR AUKE BAY AK | 1998 | 2004 | 11.2 | 7.3 | 6.7 | 9.1 | 12.8 | 10.7 | 7.5 | 8.3 | 16.8 | 19.8 | 12.6 | 16.3 | 11.6 |
| REYNOLDS C NR HYDABURG AK | 1982 | 2003 | 94.5 | 70.6 | 56.0 | 60.5 | 76.9 | 64.6 | 46.1 | 52.3 | 70.8 | 97.3 | 80.2 | 73.9 | 70.3 |
| ROCKY PASS C NR POINT BAKER AK | 1977 | 1988 | 18.1 | 14.8 | 12.7 | 10.0 | 6.7 | 5.0 | 5.1 | 8.1 | 16.5 | 29.4 | 19.1 | 15.8 | 13.4 |
| SILVER BAY TR NR SITKA AK | 1999 | 2011 | 2.6 | 1.8 | 1.4 | 2.5 | 4.1 | 2.7 | 2.1 | 2.5 | 5.4 | 6.0 | 4.3 | 3.6 | 3.3 |
| SITUK R NR YAKUTAT AK | 1988 | 2011 | 273.5 | 255.3 | 213.3 | 220.2 | 266.0 | 208.1 | 181.7 | 284.1 | 479.5 | 530.5 | 356.1 | 376.2 | 303.7 |
| SKAGWAY R AT SKAGWAY AK | 1977 | 1986 | 60.9 | 44.4 | 38.7 | 88.9 | 597.9 | 1684.8 | 1961.1 | 1489.2 | 756.9 | 634.0 | 109.5 | 55.4 | 626.8 |
| STANEY C NR KLAWOCK AK | 1989 | 2011 | 475.6 | 375.8 | 346.3 | 309.1 | 228.4 | 117.4 | 116.0 | 201.4 | 526.4 | 690.0 | 575.2 | 565.3 | 377.2 |
| STIKINE R NR WRANGELL AK | 1977 | 2011 | 11143.4 | 9014.5 | 9749.9 | 16548.1 | 68139.4 | 136488.6 | 133065.7 | 105460.6 | 81642.3 | 55924.4 | 24068.5 | 14275.7 | 55460.1 |
| SUNRISE LK NR WRANGELL AK | 1977 | 2001 | 6.0 | 3.5 | 3.8 | 6.7 | 17.4 | 20.9 | 14.9 | 10.1 | 11.6 | 17.7 | 9.0 | 8.5 | 10.8 |
| TAIYA R NR SKAGWAY AK | 1977 | 2011 | 101.0 | 105.0 | 89.0 | 252.0 | 1318.0 | 2712.0 | 3357.0 | 3276.0 | 1999.0 | 846.0 | 343.0 | 183.0 | 1215.1 |
| TAKU R NR JUNEAU AK | 1987 | 2011 | 2224.8 | 1896.8 | 2286.0 | 4199.3 | 20447.6 | 34870.8 | 31565.4 | 26019.2 | 19072.1 | 11612.0 | 5204.5 | 3362.2 | 13563.4 |
| THREEMILE C NR KLAWOCK AK | 1999 | 2003 | 58.9 | 32.4 | 32.3 | 38.4 | 68.9 | 72.5 | 53.8 | 55.2 | 91.8 | 81.1 | 62.1 | 58.4 | 58.8 |
| TONALITE C NR TENAKEE AK | 1977 | 1988 | 102.0 | 84.0 | 62.0 | 97.0 | 134.0 | 85.0 | 43.0 | 50.0 | 98.0 | 234.9 | 116.1 | 87.2 | 99.4 |
| WHITE C NR KETCHIKAN AK | 1977 | 1984 | 16.0 | 13.0 | 14.0 | 22.0 | 42.0 | 43.0 | 25.0 | 22.0 | 31.0 | 52.0 | 25.0 | 16.0 | 26.8 |
|  |  |  |  |  |  |  |  |  |  |  |  |  |  |  |  |
| Monthly Mean |  |  | 562.4 | 460.8 | 474.4 | 784.7 | 3089.9 | 6357.9 | 6701.3 | 5611.0 | 4042.9 | 2614.6 | 1148.2 | 727.8 | 2714.7 |
|  |  |  |  |  |  |  |  |  |  |  |  |  |  |  |  |
