# Supplementary material for: Climate Change Sensitivity Index for Pacific Salmon Habitat in Southeast Alaska
Source: PLoS One. 2014 Aug 15;9(8):e104799. doi: 10.1371/journal.pone.0104799 (PMC4134244; doi:10.1371/journal.pone.0104799)
Supplement: Table S2 — Multiple regression-based monthly discharge model predictions for 41 southeast, Alaska, USA gauge station catchments. (DOCX) [file pone.0104799.s002.docx]

**Table S2.** Multiple regression-based monthly discharge model predictions for 41 southeast, Alaska, USA gauge station catchments.

|  | Month | | | | | | | | | | | | Yearly Mean |
| --- | --- | --- | --- | --- | --- | --- | --- | --- | --- | --- | --- | --- | --- |
| Gauge Station | JAN | FEB | MAR | APR | MAY | JUN | JUL | AUG | SEP | OCT | NOV | DEC |  |
|  |  |  |  |  |  |  |  |  |  |  |  |  |  |
| ALSEK R NR YAKUTAT AK | 3462.8 | 3237.2 | 2335.3 | 5698.8 | 38193.5 | 97904.0 | 99290.7 | 85716.7 | 50792.6 | 24688.4 | 7878.8 | 4659.2 | 35321.5 |
| ANTLER R NR AUKE BAY AK | 81.6 | 87.0 | 55.7 | 101.0 | 271.1 | 529.7 | 550.1 | 523.4 | 470.6 | 342.8 | 135.6 | 80.3 | 269.1 |
| BIG C NR POINT BAKER AK | 78.6 | 172.6 | 68.9 | 77.2 | 69.5 | 48.4 | 34.6 | 38.0 | 81.5 | 155.3 | 123.8 | 90.4 | 86.6 |
| BLACK R NR PELICAN AK | 185.1 | 286.5 | 139.1 | 153.2 | 193.9 | 152.3 | 136.9 | 159.3 | 347.9 | 552.2 | 289.0 | 183.8 | 231.6 |
| DOROTHY LK OUTLET NR JUNEAU AK | 37.3 | 67.7 | 30.2 | 30.0 | 117.9 | 252.7 | 303.6 | 223.0 | 208.4 | 182.1 | 64.0 | 44.5 | 130.1 |
| DUCK C BL NANCY ST NR AUKE BAY AK | 5.0 | 12.3 | 4.2 | 4.1 | 2.7 | 1.1 | 1.0 | 2.4 | 5.8 | 12.1 | 7.7 | 6.6 | 5.4 |
| FARRAGUT R NR PETERSBURG AK | 445.2 | 708.1 | 451.8 | 624.4 | 1551.3 | 2085.3 | 2021.2 | 1585.7 | 1905.7 | 1849.0 | 745.8 | 513.2 | 1207.2 |
| FISH C NR KETCHIKAN AK | 305.0 | 855.0 | 280.4 | 368.1 | 408.7 | 366.9 | 229.0 | 262.7 | 416.4 | 712.1 | 399.7 | 300.7 | 408.7 |
| GOAT C NR WRANGELL AK | 47.8 | 99.8 | 48.0 | 59.7 | 187.7 | 238.7 | 193.3 | 152.6 | 190.0 | 218.1 | 75.1 | 67.2 | 131.5 |
| GOAT LK OUTLET NR SKAGWAY AK | 1.6 | 3.6 | 1.6 | 2.3 | 17.5 | 42.1 | 38.9 | 27.5 | 17.8 | 9.8 | 3.3 | 2.9 | 14.1 |
| GOLD C NR JUNEAU AK | 30.8 | 54.0 | 23.5 | 40.0 | 114.9 | 114.3 | 102.2 | 97.5 | 130.4 | 114.5 | 57.9 | 38.5 | 76.5 |
| HARDING R NR WRANGELL AK | 250.9 | 463.7 | 260.7 | 342.8 | 921.0 | 885.4 | 719.0 | 646.5 | 845.2 | 1180.6 | 636.0 | 378.9 | 627.6 |
| INDIAN R NR SITKA AK | 85.2 | 156.3 | 52.9 | 60.6 | 81.4 | 81.4 | 60.6 | 63.4 | 126.9 | 171.1 | 101.5 | 80.3 | 93.5 |
| INDIAN R NR TENAKEE AK | 54.8 | 119.5 | 48.4 | 63.2 | 101.4 | 84.5 | 80.9 | 77.0 | 193.2 | 170.0 | 124.1 | 71.0 | 99.0 |
| KAHTAHEENA R NR GUSTAVUS AK | 36.7 | 66.9 | 26.8 | 44.9 | 83.9 | 83.3 | 74.5 | 72.1 | 119.3 | 129.1 | 67.2 | 45.5 | 70.9 |
| KAKUHAN C NR HAINES AK | 0.6 | 1.8 | 0.3 | 2.5 | 16.9 | 34.1 | 38.2 | 35.8 | 27.6 | 12.6 | 4.3 | 2.0 | 14.7 |
| KETA R NR KETCHIKAN AK | 525.6 | 963.2 | 321.6 | 676.0 | 1245.5 | 1435.7 | 969.0 | 982.9 | 1022.3 | 1588.3 | 596.1 | 444.4 | 897.5 |
| KLEHINI R NR KLUKWAN AK | 175.9 | 227.2 | 123.3 | 288.8 | 1175.1 | 2228.3 | 2084.0 | 2116.7 | 1927.4 | 1251.3 | 427.4 | 257.9 | 1023.6 |
| LEMON C NR JUNEAU AK | 22.6 | 37.0 | 15.6 | 38.5 | 147.1 | 303.2 | 396.6 | 473.8 | 439.8 | 173.7 | 76.2 | 32.6 | 179.7 |
| MAHONEY C NR KETCHIKAN AK | 53.2 | 123.5 | 39.5 | 49.9 | 77.9 | 86.4 | 51.2 | 53.4 | 79.1 | 116.0 | 63.0 | 50.7 | 70.3 |
| MENDENHALL R NR AUKE BAY AK | 126.3 | 207.4 | 102.4 | 217.3 | 778.0 | 1735.8 | 3555.4 | 3570.2 | 1688.3 | 1098.3 | 420.1 | 194.6 | 1141.2 |
| MONTANA C NR AUKE BAY AK | 39.7 | 67.7 | 31.5 | 43.2 | 99.0 | 127.7 | 145.2 | 131.9 | 115.5 | 147.6 | 69.4 | 51.1 | 89.1 |
| NAKWASINA R NR SITKA AK | 249.7 | 404.4 | 156.6 | 176.1 | 322.7 | 360.7 | 314.0 | 275.4 | 526.5 | 631.6 | 352.2 | 263.7 | 336.1 |
| OLD TOM C NR KASAAN AK | 50.0 | 100.6 | 32.3 | 46.9 | 52.3 | 35.0 | 21.5 | 22.4 | 47.9 | 82.4 | 76.1 | 62.0 | 52.5 |
| OPHIR C NR YAKUTAT AK | 21.7 | 39.7 | 13.3 | 10.1 | 8.8 | 4.8 | 4.5 | 10.0 | 21.9 | 39.0 | 21.8 | 25.6 | 18.4 |
| PAVLOF R NR TENAKEE AK | 118.9 | 254.3 | 125.7 | 159.5 | 173.3 | 118.5 | 84.4 | 105.6 | 225.1 | 273.9 | 187.0 | 122.3 | 162.4 |
| PERKINS C NR METLAKATLA | 40.6 | 80.3 | 25.6 | 34.6 | 30.3 | 20.7 | 11.8 | 13.9 | 32.5 | 64.0 | 48.9 | 38.7 | 36.8 |
| PETERSON C BL NF NR AUKE BAY AK | 13.9 | 25.6 | 10.5 | 17.3 | 27.0 | 21.9 | 16.5 | 18.7 | 32.1 | 37.7 | 21.3 | 15.3 | 21.5 |
| REYNOLDS C NR HYDABURG AK | 77.8 | 144.6 | 51.3 | 58.1 | 75.0 | 69.1 | 47.3 | 43.9 | 69.2 | 117.0 | 93.0 | 73.2 | 76.6 |
| ROCKY PASS C NR POINT BAKER AK | 19.1 | 40.2 | 13.5 | 13.4 | 10.9 | 7.5 | 5.7 | 7.4 | 15.0 | 32.7 | 26.6 | 20.4 | 17.7 |
| SILVER BAY TR NR SITKA AK | 2.7 | 4.9 | 0.9 | 1.1 | 3.3 | 4.1 | 2.6 | 2.5 | 5.4 | 6.1 | 2.9 | 2.6 | 3.3 |
| SITUK R NR YAKUTAT AK | 204.9 | 423.6 | 185.6 | 183.9 | 264.4 | 202.9 | 183.7 | 277.9 | 480.5 | 594.7 | 257.6 | 258.8 | 293.2 |
| SKAGWAY R AT SKAGWAY AK | 94.9 | 131.0 | 78.5 | 127.3 | 685.1 | 1749.2 | 1978.7 | 1582.2 | 878.9 | 499.9 | 212.4 | 141.2 | 679.9 |
| STANEY C NR KLAWOCK AK | 485.6 | 832.1 | 320.9 | 513.7 | 395.4 | 260.9 | 183.6 | 199.0 | 429.1 | 874.3 | 714.9 | 507.6 | 476.4 |
| STIKINE R NR WRANGELL AK | 10643.5 | 12789.6 | 8077.3 | 12707.7 | 68104.1 | 158864.1 | 174622.3 | 132453.3 | 83367.2 | 55194.7 | 20349.7 | 11401.1 | 62381.2 |
| SUNRISE LK NR WRANGELL AK | 4.9 | 10.7 | 3.1 | 3.7 | 13.0 | 13.6 | 9.8 | 7.2 | 10.8 | 13.7 | 5.3 | 3.9 | 8.3 |
| TAIYA R NR SKAGWAY AK | 107.4 | 122.3 | 72.9 | 207.3 | 858.1 | 1987.2 | 1976.1 | 1994.0 | 1464.2 | 725.2 | 276.5 | 150.4 | 828.5 |
| TAKU R NR JUNEAU AK | 4347.5 | 4371.3 | 3370.6 | 4728.7 | 21661.9 | 44708.1 | 47102.1 | 36549.5 | 27479.3 | 19729.9 | 7252.0 | 4786.1 | 18840.6 |
| THREEMILE C NR KLAWOCK AK | 68.1 | 89.8 | 32.4 | 51.0 | 77.5 | 77.7 | 51.6 | 52.1 | 84.8 | 134.7 | 101.4 | 93.8 | 76.2 |
| TONALITE C NR TENAKEE AK | 71.1 | 134.6 | 64.5 | 73.0 | 94.0 | 77.9 | 57.1 | 62.7 | 135.4 | 166.2 | 108.4 | 72.6 | 93.1 |
| WHITE C NR KETCHIKAN AK | 16.3 | 27.9 | 8.3 | 16.2 | 37.8 | 48.0 | 31.3 | 27.9 | 31.7 | 42.2 | 16.6 | 14.3 | 26.5 |
|  |  |  |  |  |  |  |  |  |  |  |  |  |  |
| Monthly Mean | 553.4 | 684.0 | 417.2 | 685.8 | 3384.2 | 7742.8 | 8238.6 | 6602.9 | 4304.6 | 2783.8 | 1036.4 | 625.6 | 3088.3 |
|  |  |  |  |  |  |  |  |  |  |  |  |  |  |
