# Supplementary material for: Climate Change Sensitivity Index for Pacific Salmon Habitat in Southeast Alaska
Source: PLoS One. 2014 Aug 15;9(8):e104799. doi: 10.1371/journal.pone.0104799 (PMC4134244; doi:10.1371/journal.pone.0104799)
Supplement: Table S5 — Multiple regression-based monthly discharge models for projected future discharge (cubic feet per second) for 41 gauged catchments in southeast, Alaska, USA using an ensemble global climate model average (ECHAM5, HadCM3, CGCM3.1) and the A2 global greenhouse gas emission scenario for the year 2080. (DOCX) [file pone.0104799.s005.docx]

**Table S5.** Multiple regression-based monthly discharge models for projected future discharge (cubic feet per second) for 41 gauged catchments in southeast, Alaska, USA using an ensemble global climate model average (ECHAM5, HadCM3, CGCM3.1) and the A2 global greenhouse gas emission scenario for the year 2080.

|  | Month | | | | | | | | | | | | Yearly Mean |
| --- | --- | --- | --- | --- | --- | --- | --- | --- | --- | --- | --- | --- | --- |
| Gauge Station | JAN | FEB | MAR | APR | MAY | JUN | JUL | AUG | SEP | OCT | NOV | DEC |  |
|  |  |  |  |  |  |  |  |  |  |  |  |  |  |
| ALSEK R NR YAKUTAT AK | 7291.6 | 10610.6 | 5072.6 | 15446.6 | 70074.0 | 102150.7 | 56250.0 | 65548.9 | 63687.7 | 48119.7 | 18509.9 | 8407.1 | 39264.1 |
| ANTLER R NR AUKE BAY AK | 222.0 | 353.2 | 147.0 | 459.2 | 776.9 | 600.6 | 366.7 | 446.3 | 461.7 | 780.2 | 400.3 | 165.2 | 431.6 |
| BIG C NR POINT BAKER AK | 169.1 | 524.8 | 166.1 | 305.0 | 146.2 | 48.4 | 21.3 | 29.2 | 94.2 | 301.3 | 274.7 | 152.9 | 186.1 |
| BLACK R NR PELICAN AK | 398.5 | 931.7 | 331.7 | 632.2 | 446.9 | 155.3 | 94.6 | 131.0 | 352.6 | 1122.0 | 719.9 | 335.0 | 470.9 |
| DOROTHY LK OUTLET NR JUNEAU AK | 100.0 | 279.1 | 80.9 | 143.4 | 340.4 | 281.7 | 201.8 | 191.6 | 195.3 | 434.8 | 189.1 | 90.9 | 210.7 |
| DUCK C BL NANCY ST NR AUKE BAY AK | 11.8 | 43.5 | 11.0 | 18.3 | 7.7 | 1.2 | 0.3 | 1.7 | 6.8 | 24.5 | 19.5 | 12.6 | 13.2 |
| FARRAGUT R NR PETERSBURG AK | 1153.6 | 2586.5 | 1216.6 | 2849.3 | 4205.8 | 2278.5 | 1309.2 | 1292.9 | 1957.6 | 4100.2 | 2068.5 | 972.6 | 2165.9 |
| FISH C NR KETCHIKAN AK | 751.5 | 3019.5 | 775.7 | 1810.7 | 930.5 | 366.5 | 129.9 | 194.0 | 435.8 | 1528.4 | 936.7 | 519.2 | 949.9 |
| GOAT C NR WRANGELL AK | 127.6 | 358.9 | 133.7 | 273.8 | 500.8 | 251.9 | 118.2 | 119.5 | 201.8 | 500.2 | 200.6 | 127.5 | 242.9 |
| GOAT LK OUTLET NR SKAGWAY AK | 4.4 | 14.2 | 4.7 | 9.1 | 40.1 | 43.8 | 23.7 | 21.3 | 21.5 | 20.5 | 9.3 | 6.0 | 18.2 |
| GOLD C NR JUNEAU AK | 72.6 | 204.8 | 60.6 | 176.1 | 317.3 | 122.1 | 66.7 | 81.1 | 133.3 | 242.9 | 152.3 | 74.2 | 142.0 |
| HARDING R NR WRANGELL AK | 693.4 | 1748.1 | 773.4 | 1741.0 | 2566.5 | 929.1 | 432.2 | 503.4 | 866.2 | 2846.3 | 1980.8 | 729.5 | 1317.5 |
| INDIAN R NR SITKA AK | 180.6 | 527.7 | 127.2 | 258.8 | 180.8 | 81.8 | 40.0 | 50.9 | 134.9 | 328.3 | 224.8 | 140.5 | 189.7 |
| INDIAN R NR TENAKEE AK | 118.2 | 420.6 | 117.0 | 278.1 | 233.6 | 84.5 | 54.3 | 61.6 | 197.0 | 334.3 | 315.4 | 131.1 | 195.5 |
| KAHTAHEENA R NR GUSTAVUS AK | 81.7 | 245.8 | 63.8 | 181.2 | 207.7 | 85.7 | 47.9 | 57.8 | 124.4 | 265.9 | 174.4 | 86.5 | 135.2 |
| KAKUHAN C NR HAINES AK | 2.6 | 10.3 | 2.1 | 12.8 | 49.6 | 38.4 | 25.1 | 30.2 | 27.8 | 28.8 | 13.9 | 4.9 | 20.5 |
| KETA R NR KETCHIKAN AK | 1454.8 | 3673.1 | 913.8 | 3766.5 | 3033.8 | 1468.3 | 539.1 | 724.2 | 1029.6 | 3735.5 | 1502.8 | 805.6 | 1887.3 |
| KLEHINI R NR KLUKWAN AK | 370.8 | 788.2 | 275.9 | 864.5 | 2322.1 | 2192.9 | 1186.8 | 1600.3 | 2373.0 | 2495.7 | 1048.3 | 477.5 | 1333.0 |
| LEMON C NR JUNEAU AK | 56.9 | 151.0 | 41.9 | 179.7 | 425.2 | 342.5 | 277.2 | 425.2 | 397.8 | 388.3 | 216.5 | 65.6 | 247.3 |
| MAHONEY C NR KETCHIKAN AK | 132.9 | 432.8 | 111.5 | 255.7 | 178.3 | 86.2 | 29.0 | 39.6 | 79.4 | 253.0 | 149.1 | 87.6 | 152.9 |
| MENDENHALL R NR AUKE BAY AK | 313.4 | 853.4 | 266.7 | 987.9 | 2161.0 | 1931.6 | 2518.9 | 3171.1 | 1714.8 | 2427.6 | 1159.2 | 393.3 | 1491.6 |
| MONTANA C NR AUKE BAY AK | 90.5 | 245.6 | 77.3 | 177.2 | 244.0 | 133.8 | 94.4 | 107.7 | 129.3 | 301.6 | 174.6 | 96.2 | 156.0 |
| NAKWASINA R NR SITKA AK | 564.3 | 1483.8 | 395.7 | 823.8 | 777.5 | 368.3 | 212.3 | 225.1 | 522.2 | 1277.5 | 857.2 | 484.3 | 666.0 |
| OLD TOM C NR KASAAN AK | 106.2 | 296.6 | 79.1 | 186.8 | 105.3 | 34.2 | 12.5 | 16.5 | 54.8 | 159.0 | 164.0 | 102.4 | 109.8 |
| OPHIR C NR YAKUTAT AK | 52.1 | 156.7 | 32.7 | 36.8 | 21.2 | 5.9 | 2.7 | 9.0 | 20.7 | 85.6 | 59.7 | 52.4 | 44.6 |
| PAVLOF R NR TENAKEE AK | 255.1 | 864.1 | 298.0 | 656.6 | 388.5 | 118.1 | 54.1 | 82.8 | 252.9 | 523.0 | 431.0 | 217.7 | 345.1 |
| PERKINS C NR METLAKATLA | 88.2 | 242.2 | 66.0 | 148.4 | 59.5 | 20.0 | 6.5 | 9.9 | 35.4 | 126.1 | 102.6 | 62.5 | 80.6 |
| PETERSON C BL NF NR AUKE BAY AK | 31.0 | 88.8 | 26.3 | 72.8 | 65.0 | 22.5 | 10.2 | 14.6 | 36.6 | 75.0 | 51.8 | 28.4 | 43.6 |
| REYNOLDS C NR HYDABURG AK | 195.1 | 478.6 | 141.7 | 292.8 | 165.4 | 69.0 | 28.2 | 33.3 | 69.1 | 253.5 | 227.8 | 124.4 | 173.2 |
| ROCKY PASS C NR POINT BAKER AK | 41.1 | 126.7 | 33.0 | 53.8 | 23.9 | 7.4 | 3.2 | 5.6 | 18.0 | 62.8 | 59.8 | 35.3 | 39.2 |
| SILVER BAY TR NR SITKA AK | 7.1 | 20.1 | 3.6 | 8.4 | 9.1 | 4.1 | 1.4 | 1.9 | 5.5 | 13.1 | 8.0 | 5.4 | 7.3 |
| SITUK R NR YAKUTAT AK | 503.8 | 1743.5 | 451.2 | 660.7 | 623.8 | 247.3 | 123.7 | 255.6 | 443.6 | 1332.5 | 713.9 | 533.8 | 636.1 |
| SKAGWAY R AT SKAGWAY AK | 201.5 | 442.4 | 176.4 | 393.1 | 1543.6 | 1837.0 | 1232.5 | 1253.7 | 1041.8 | 1011.7 | 526.5 | 257.6 | 826.5 |
| STANEY C NR KLAWOCK AK | 1055.7 | 2512.7 | 783.7 | 2097.7 | 823.4 | 260.9 | 113.9 | 152.9 | 476.4 | 1736.5 | 1602.5 | 849.4 | 1038.8 |
| STIKINE R NR WRANGELL AK | 22671.2 | 36255.3 | 17235.4 | 36109.4 | 142307.1 | 158041.3 | 97822.5 | 97680.2 | 107881.9 | 105131.7 | 46039.0 | 19586.0 | 73896.7 |
| SUNRISE LK NR WRANGELL AK | 12.9 | 36.4 | 9.2 | 19.1 | 33.0 | 13.9 | 5.6 | 5.3 | 12.0 | 30.1 | 13.4 | 7.5 | 16.5 |
| TAIYA R NR SKAGWAY AK | 222.9 | 401.0 | 161.2 | 607.5 | 1846.4 | 2056.2 | 1199.2 | 1552.6 | 1779.1 | 1439.8 | 668.9 | 270.5 | 1017.1 |
| TAKU R NR JUNEAU AK | 8861.3 | 12441.0 | 7121.6 | 13327.8 | 44508.1 | 43559.8 | 27005.0 | 26827.9 | 35903.3 | 36493.6 | 16241.9 | 8271.7 | 23380.3 |
| THREEMILE C NR KLAWOCK AK | 157.4 | 285.5 | 84.0 | 227.2 | 168.7 | 78.5 | 31.5 | 40.4 | 86.2 | 290.5 | 249.3 | 162.8 | 155.2 |
| TONALITE C NR TENAKEE AK | 152.2 | 453.7 | 154.2 | 300.3 | 205.8 | 77.6 | 36.9 | 49.0 | 151.2 | 317.6 | 246.9 | 128.3 | 189.5 |
| WHITE C NR KETCHIKAN AK | 45.2 | 104.8 | 25.3 | 89.6 | 90.4 | 48.5 | 16.9 | 20.1 | 32.6 | 97.4 | 42.7 | 26.4 | 53.3 |
|  |  |  |  |  |  |  |  |  |  |  |  |  |  |
| Monthly Mean | 1195.7 | 2108.7 | 928.0 | 2120.5 | 6906.2 | 7818.2 | 4676.7 | 4952.8 | 5449.9 | 5391.2 | 2408.5 | 1099.7 | 3754.7 |
|  |  |  |  |  |  |  |  |  |  |  |  |  |  |
