# Supplementary material for: Climate Change Sensitivity Index for Pacific Salmon Habitat in Southeast Alaska
Source: PLoS One. 2014 Aug 15;9(8):e104799. doi: 10.1371/journal.pone.0104799 (PMC4134244; doi:10.1371/journal.pone.0104799)
Supplement: Table S6 — Multiple regression-based monthly discharge model percent error ((predicted-observed)/observed) for 41 southeast, Alaska, USA gauged catchments. (DOCX) [file pone.0104799.s006.docx]

**Table S6.** Multiple regression-based monthly discharge model percent error ([predicted-observed]/observed) for 41 southeast, Alaska, USA gauged catchments.

|  |  |  |  |  |  | Month |  |  |  |  |  |  | Yearly \|Mean\| |
| --- | --- | --- | --- | --- | --- | --- | --- | --- | --- | --- | --- | --- | --- |
| Gauge Stations | JAN | FEB | MAR | APR | MAY | JUN | JUL | AUG | SEP | OCT | NOV | DEC |  |
| ALSEK R NR YAKUTAT AK | -30.6% | -21.1% | -40.5% | -11.9% | 46.1% | 39.6% | 11.4% | 8.4% | 6.4% | 3.5% | -24.1% | -33.7% | 23.1% |
| ANTLER R NR AUKE BAY AK | 125.2% | 201.5% | 121.9% | 122.5% | 63.4% | 75.3% | 111.2% | 137.0% | 119.5% | 115.5% | 75.4% | 38.2% | 108.9% |
| BIG C NR POINT BAKER AK | 15.8% | 109.3% | -2.7% | -16.2% | 23.2% | 8.2% | 23.4% | -7.8% | -18.6% | -38.8% | -17.5% | 12.3% | 24.5% |
| BLACK R NR PELICAN AK | 22.6% | 116.1% | -12.6% | -35.9% | -42.9% | -31.2% | -26.0% | 1.1% | 21.3% | -4.1% | 5.3% | 29.9% | 29.1% |
| DOROTHY LK OUTLET NR JUNEAU AK | 73.3% | 227.7% | 74.7% | 59.2% | 36.8% | 16.2% | 12.0% | -15.4% | -20.1% | 10.9% | 29.1% | 18.2% | 49.5% |
| DUCK C BL NANCY ST NR AUKE BAY AK | 58.1% | 352.3% | 67.4% | 46.5% | 9.7% | -45.2% | -61.0% | -34.2% | -23.9% | 33.6% | 59.0% | 24.2% | 67.9% |
| FARRAGUT R NR PETERSBURG AK | -44.0% | 9.2% | -19.4% | -17.3% | -9.6% | -15.4% | -27.7% | -43.0% | -33.2% | -28.6% | -36.4% | -30.0% | 26.1% |
| FISH C NR KETCHIKAN AK | -24.5% | 141.1% | -7.7% | 1.5% | -10.7% | -10.2% | -23.4% | -15.6% | -19.2% | 5.2% | -24.5% | -32.3% | 26.3% |
| GOAT C NR WRANGELL AK | -30.0% | 91.0% | -3.0% | -23.3% | -31.1% | -38.6% | -38.3% | -36.2% | -31.2% | -38.1% | -29.3% | 28.9% | 34.9% |
| GOAT LK OUTLET NR SKAGWAY AK | 7.5% | 98.2% | 4.8% | 62.0% | -8.0% | -2.6% | 22.9% | 29.4% | -1.2% | -10.1% | 7.0% | 55.5% | 25.8% |
| GOLD C NR JUNEAU AK | 22.8% | 116.9% | 7.0% | -3.9% | -17.3% | -42.9% | -43.9% | -39.8% | -27.3% | -28.6% | -1.6% | -12.3% | 30.4% |
| HARDING R NR WRANGELL AK | -20.9% | 84.1% | 12.0% | -17.8% | -2.2% | -36.0% | -43.2% | -40.4% | -30.4% | 8.6% | 24.9% | 7.1% | 27.3% |
| INDIAN R NR SITKA AK | -9.8% | 96.7% | -11.2% | -8.4% | -18.3% | -1.2% | -2.4% | -26.5% | -25.9% | -4.1% | -12.9% | -24.7% | 20.2% |
| INDIAN R NR TENAKEE AK | -5.8% | 177.2% | 21.1% | -17.8% | -28.1% | -23.2% | 39.5% | 113.9% | 103.4% | -3.4% | 17.1% | 24.6% | 47.9% |
| KAHTAHEENA R NR GUSTAVUS AK | 5.0% | 147.6% | 41.3% | 32.1% | 1.1% | 1.6% | 35.4% | 36.1% | 20.5% | 46.7% | 24.4% | -18.7% | 34.2% |
| KAKUHAN C NR HAINES AK | -55.3% | 80.8% | -69.8% | 4.7% | 68.9% | 62.3% | 36.4% | 37.7% | 53.5% | 30.2% | 7.7% | -22.0% | 44.1% |
| KETA R NR KETCHIKAN AK | 28.6% | 228.2% | 5.4% | 44.3% | 8.5% | 17.8% | 13.5% | 27.2% | 6.7% | 7.4% | -16.0% | 2.4% | 33.8% |
| KLEHINI R NR KLUKWAN AK | -38.3% | -6.2% | -44.5% | -25.9% | -34.1% | -40.3% | -49.8% | -33.9% | 9.3% | -5.4% | -17.4% | -31.3% | 28.0% |
| LEMON C NR JUNEAU AK | 38.3% | 263.8% | 67.2% | 25.5% | 10.9% | -1.5% | -14.0% | -6.6% | -0.8% | -9.2% | 32.5% | 31.4% | 41.8% |
| MAHONEY C NR KETCHIKAN AK | -7.6% | 117.3% | -36.9% | -23.5% | -29.2% | -29.1% | -41.2% | -21.3% | -25.9% | -40.1% | -43.3% | -25.2% | 36.7% |
| MENDENHALL R NR AUKE BAY AK | -6.8% | 107.3% | 5.6% | 33.5% | 2.1% | -13.7% | 14.0% | 4.8% | -38.2% | -23.9% | 24.1% | 1.0% | 22.9% |
| MONTANA C NR AUKE BAY AK | -38.0% | 56.4% | -17.4% | -22.3% | -15.9% | -8.8% | 9.6% | -8.1% | -34.4% | -4.6% | -15.7% | -17.8% | 20.8% |
| NAKWASINA R NR SITKA AK | 60.1% | 189.0% | 56.1% | 15.4% | -3.0% | -23.9% | -19.4% | -6.2% | 23.6% | 0.6% | 18.2% | 68.7% | 40.3% |
| OLD TOM C NR KASAAN AK | -21.3% | 88.5% | -36.8% | -10.0% | 36.7% | 60.0% | 81.1% | 35.2% | 26.3% | 12.4% | -0.8% | -10.4% | 35.0% |
| OPHIR C NR YAKUTAT AK | 19.2% | 159.7% | -0.6% | -20.1% | -30.9% | -17.6% | 13.0% | 14.7% | 14.2% | 26.6% | -13.6% | 11.7% | 28.5% |
| PAVLOF R NR TENAKEE AK | -18.7% | 107.5% | 17.2% | -11.6% | -12.7% | -20.0% | -26.1% | 17.1% | 4.9% | -46.0% | -10.9% | 55.2% | 29.0% |
| PERKINS C NR METLAKATLA | -23.5% | 87.4% | -37.0% | -2.4% | 24.6% | 43.5% | 4.0% | -11.4% | -3.3% | 10.8% | -29.5% | -23.3% | 25.0% |
| PETERSON C BL NF NR AUKE BAY AK | 24.4% | 251.0% | 56.2% | 90.8% | 111.5% | 105.2% | 120.2% | 124.4% | 91.5% | 90.6% | 69.7% | -6.1% | 95.1% |
| REYNOLDS C NR HYDABURG AK | -17.7% | 104.7% | -8.3% | -4.0% | -2.4% | 7.0% | 2.5% | -16.0% | -2.3% | 20.2% | 16.0% | -1.0% | 16.8% |
| ROCKY PASS C NR POINT BAKER AK | 5.5% | 171.2% | 5.6% | 33.9% | 62.0% | 49.0% | 11.8% | -8.3% | -9.3% | 11.3% | 39.3% | 29.2% | 36.4% |
| SILVER BAY TR NR SITKA AK | 1.9% | 168.2% | -41.0% | -58.0% | -18.1% | 50.6% | 21.7% | 2.6% | -1.2% | 1.9% | -32.3% | -29.6% | 35.6% |
| SITUK R NR YAKUTAT AK | -25.1% | 65.9% | -13.0% | -16.5% | -0.6% | -2.5% | 1.1% | -2.2% | 0.2% | 12.1% | -27.7% | -31.2% | 16.5% |
| SKAGWAY R AT SKAGWAY AK | 55.9% | 195.1% | 103.1% | 43.3% | 14.6% | 3.8% | 0.9% | 6.2% | 16.1% | -21.2% | 94.0% | 155.0% | 59.1% |
| STANEY C NR KLAWOCK AK | 2.1% | 121.4% | -7.3% | 66.2% | 73.1% | 122.2% | 58.3% | -1.2% | -18.5% | 26.7% | 24.3% | -10.2% | 44.3% |
| STIKINE R NR WRANGELL AK | -4.5% | 41.9% | -17.2% | -23.2% | -0.1% | 16.4% | 31.2% | 25.6% | 2.1% | -1.3% | -15.5% | -20.1% | 16.6% |
| SUNRISE LK NR WRANGELL AK | -17.7% | 208.3% | -17.8% | -44.4% | -25.2% | -35.3% | -34.4% | -28.8% | -7.6% | -22.9% | -41.5% | -53.6% | 44.8% |
| TAIYA R NR SKAGWAY AK | 6.3% | 16.5% | -18.1% | -17.7% | -34.9% | -26.7% | -41.1% | -39.1% | -26.8% | -14.3% | -19.4% | -17.8% | 23.2% |
| TAKU R NR JUNEAU AK | 95.4% | 130.5% | 47.4% | 12.6% | 5.9% | 28.2% | 49.2% | 40.5% | 44.1% | 69.9% | 39.3% | 42.4% | 50.5% |
| THREEMILE C NR KLAWOCK AK | 15.6% | 177.5% | 0.5% | 32.9% | 12.4% | 7.2% | -4.0% | -5.7% | -7.6% | 66.2% | 63.3% | 60.7% | 37.8% |
| TONALITE C NR TENAKEE AK | -30.3% | 60.3% | 4.0% | -24.7% | -29.9% | -8.3% | 32.7% | 25.3% | 38.2% | -29.2% | -6.6% | -16.7% | 25.5% |
| WHITE C NR KETCHIKAN AK | 1.8% | 114.6% | -40.5% | -26.5% | -10.1% | 11.7% | 25.0% | 26.9% | 2.2% | -18.9% | -33.4% | -10.4% | 26.8% |
|  |  |  |  |  |  |  |  |  |  |  |  |  |  |
| Monthly \|Mean\| | 28.2% | 129.5% | 29.8% | 29.5% | 25.0% | 29.3% | 31.2% | 28.3% | 24.7% | 24.5% | 27.8% | 28.7% | 36.4% |
|  |  |  |  |  |  |  |  |  |  |  |  |  |  |
